# Access to maternal health services during COVID-19 pandemic, re-examining the three delays among pregnant women in Ilubabor zone, southwest Ethiopia: A cross-sectional study

**DOI:** 10.1371/journal.pone.0268196

**Published:** 2022-05-05

**Authors:** Diriba Kumara Abdisa, Debela Dereje Jaleta, Jira Wakoya Feyisa, Keno Melkamu Kitila, Robera Demissie Berhanu

**Affiliations:** 1 Department of Health Informatics, College of Health Sciences, Mettu University, Mettu, Ethiopia; 2 Department of Nursing, College of Health Sciences, Mettu University, Mettu, Ethiopia; 3 Department of Public Health, Institute of Health Sciences, Wollega University, Nekemt, Ethiopia; 4 Department of Public Health, College of Health Sciences, Mettu University, Mettu, Ethiopia; FHI360, UNITED STATES

## Abstract

**Background:**

All women require access to high-quality care during pregnancy, labor, and after childbirth. The occurrence of delay at any stage is one of the major causes of maternal mortality. There is, however, a scarcity of data on women’s access to maternal health services during the COVID-19 pandemic. Therefore, the goal of this study was to assess the magnitude of delays in maternal health service utilization and its associated factors among pregnant women in the Ilubabor zone during the COVID-19 pandemic.

**Methods:**

A facility-based cross-sectional study was conducted among 402 pregnant women selected by systematic random sampling. Data were analyzed using IBM SPSS Statistics version 26. Descriptive and summary statistics were used to describe the study population. Bivariate and multivariable logistic regression analyses were performed to identify factors associated with the outcome variables. Adjusted odds ratio with respective 95% CI was used to report significant covariates.

**Results:**

A total of 402 pregnant women participated in this study. The median age of the respondents was 25 years (IQR = 8). On average, a woman stays 1.76 hours (SD = 1.2) to make a decision to seek care. The prevalence of first, second and third delay were 51%, 48%, and 33.3%, respectively. Being unmarried [AOR (95% CI)], [0.145 (0.046–0.452)], being unemployed [AOR (95% CI)], [4.824 (1.685–13.814)], age [AOR (95% CI)], [0.227 (0.089–0.0579)], fear of COVID-19 [AOR (95% CI)], [1.112 (1.036–1.193)], urban residence [AOR (95% CI)], [0.517 (0.295–0.909)], and lack of birth preparedness [AOR (95% CI)], [6.526 (1.954–21.789)] were significantly associated with first delay. Being unmarried [AOR (95% CI)], [5.984 (2.930–12.223)], being unemployed [AOR (95% CI)], [26.978 (3.477–209.308)], and age [AOR (95% CI)], [0.438 (0.226–0.848)] were significantly associated with second delay. Having lengthy admission [AOR (95% CI)], [7.5 (4.053–13.878)] and non-spontaneous vaginal delivery [AOR (95% CI)], [1.471 (1.018–1.999)] were significantly associated with third delay.

**Conclusion:**

This study identified a significant proportion of mothers experiencing delays, although there were no data to suggest exacerbated delays in utilizing maternal health services due to fear of the COVID-19 pandemic. The proportion of maternal delay varies with different factors. Improving the decision-making capacity of women is, therefore, essential.

## Introduction

Pregnancy and childbirth are critical times in a woman’s life since they carry major risks for both the mother and the newborn [1]. In developing countries, complication during pregnancy and childbirth is a primary cause of death and disability [2]. Nearly 75% of all maternal deaths are caused by pregnancy and delivery-related complications [3]. Access to high-quality care during pregnancy, childbirth, and afterward is very crucial to reducing maternal mortality [4]. According to Thaddeus and Maine model, there are three typical delays in accessing quality maternal care: 1) delay in deciding to seek care; 2) delay in reaching an appropriate source of care (health facility); and 3) delay in receiving adequate care [5]. Delays in utilizing maternal health services contribute to a major share of maternal mortality. A retrospective study indicated that first, second, and third maternal delays were observed in 6.3%, 50%, and 88.9% of maternal deaths, respectively [6]. The three delay model demonstrated that numerous inter-women factors lead to maternal mortality, not just a lack of economic and human resources [5].

Because of its high proportions, it is critical to pay special attention to the reduction of maternal mortality. In 2017, around 295, 000 women died as a result of pregnancy and delivery-related causes. The vast majority (94%) of these deaths occurred in areas with limited resources [7]. Despite making substantial progress in reducing maternal death over the last two decades, Ethiopia remains one of the countries considered "high alert" due to a high maternal mortality ratio in 2017 [7]. Surprisingly, prompt access to emergency obstetric interventions can save 88–98% of these deaths, making maternal mortality unique [8].

The Coronavirus Disease 2019 (COVID-19) is a global pandemic that began in late December 2019 in China and spread around the world in early 2020 [9]. More than 276 million cases and 5.3 million deaths have been reported worldwide as a result of the virus [10]. Because of its abrupt nature and the virus’s contagious potency, the pandemic poses a major threat to people’s physical and mental health [9, 11]. The international community is working to contain the spread of COVID-19 and reduce pandemic-related mortality. COVID-19 pandemic, however, may have a significant impact on maternal health services, particularly in low-income countries [12]. Evidence from previous pandemic shows that mortality indirectly attributed to the pandemic can be worse than the direct effect of the disease. The decreased use of maternal and reproductive services and an increase in maternal and neonatal death, and stillbirths indirectly caused by the epidemic outnumbered direct Ebola-related deaths [13]. Women may seek care less frequently as a result of their concern about developing COVID-19. Health-care workers who would ordinarily provide vital health services may be diverted to respond to COVID-19, resulting in fewer essential services for women [14]. Because of the shift in production to COVID-19-related supplies, worldwide supply networks and equipment may be interrupted [15]. This decline in coverage is expected to result in additional maternal deaths [16]. In low- and middle-income countries, the impact would be greater [17].

Although studies on the magnitude of delays and associated factors have been conducted in Ethiopia, studies on these conditions during the recent pandemic would be particularly useful, as the pandemic may worsen maternal delays by disrupting the health system. This study was aimed, therefore, at assessing the magnitude of delays in maternal health service utilization and its associated factors among pregnant women in the Ilubabor zone during the COVID-19 pandemic. The findings from this study will help program managers, policymakers, and other stakeholders working on maternal health to improve their understanding of current maternal health services in the area and plan interventions aimed at improving maternal health.

## Methods

### Study design, setting, and period

A facility-based cross-sectional study was conducted from February to April 2021 among pregnant women in the Ilubabor zone, which is located at about 555 km to the southwest of Ethiopia’s capital, Addis Ababa. The zone’s population is estimated to be 968,303 people, according to the 2007 Census. Of this population, 480,178 were female with roughly 214,285 of them in the reproductive age groups who gave birth to 33,600 babies in the last 12 months of the census. There are 2 hospitals and 40 health centers in the zone.

### Study population and eligibility criteria

The study population was all pregnant women who visited public health facilities in the Ilubabor zone for complications or delivery services during the study period. All pregnant women who visited the selected health facilities for pregnancy-related complications or delivery services were included in the study. Women who were severely ill and unable to respond to questions were excluded from the study.

### Sample size determination and sampling procedure

The minimum sample required for the study was calculated using Epi-info 7.2.2.2 software using the following assumptions [Table 1].

**Table 1:** Sample size calculation for respective objectives.

Objectives	Assumptions used for sample size calculation					
**Objective 1**	**Parameter**		**CI**	**d**		**N**
To determine the magnitude of delay in maternal health service utilization	Proportion of 1^st^ delay of 46.8%		95%	5%		382
	Proportion of 2^nd^ delay of 44%		95%	5%		378
	Proportion of 3^rd^ delay of 31.7%		95%	5%		333
**Objective 2**	**% outcome Unexposed**	**Ratio**	**AOR**	**CI**	**Power**	**n**
To assess factors associated with delay in maternal health service utilization	Delay among employed women of 23.2%	1:1	2.2	95%	80%	264
	Delay among well prepared women of 23.01%	1:1	2.7	95%	80%	166

CI: Confidence interval; d: Margin of error; n: Sample size; AOR: Adjusted odds ratio

Accordingly, the largest sample size was selected which was 382. After adjusting for a 10% non-response rate, the final sample size became $\underline{\underline{425}}$.

From public health facilities located in the Ilubabor zone, two hospitals and ten health centers were selected randomly using a lottery method. A systematic random sampling method was used to select the study participants in each health facility. Selected women were then interviewed until the proportionally allocated sample for that health facility was reached.

### Variables and measurement

**First maternal delay**: is the time interval between recognition of the labor and/or complication and deciding to seek a health institution. Time taken ≥1 hour to decide to seek care was considered as delay and less than an hour was considered as no delay [18].

**Second maternal delay**: is the time interval from starting to reach the health facility after the decision has been made. Time taken ≥1 hour to reach the facility was considered as delay and less than an hour was considered as no delay [18].

**Third maternal delay**: is the time interval between reaching the facility and receiving health care. Time taken ≥1 hour to receive care was considered as delay and less than an hour was considered as no delay [18].

**Fear of COVID 19 infection**: 5-point Likert scale was used and participants rated their level of agreement with the statements, ranging from “strongly disagree” to “strongly agree”. The minimum score possible for each question was 1, and the maximum was 5. A total score was calculated by adding up each item score (ranging from 7 to 35). The higher the score shows the greater the fear of COVID-19 [19].

### Study instruments and data collection

The data were collected using a structured interviewer-administered questionnaire. The questionnaire was adapted from a survey tool developed by JHPIEGO maternal and neonatal health program [20]. Fear of COVID-19 infection was assessed by using the fear of COVID-19 scale (FCV-19S) [21]. The data were collected by midwifery professionals after receiving a two-day training.

### Data quality management

The study instrument was pilot tested on 5% (22) of the sample size. Two-day training was given for data collectors regarding the objective of the study, data collection tools and procedures, how to approach respondents, and how to keep confidentiality. The collected data were checked for completeness by data collectors before leaving the respondents. Finally, 10% of the questionnaire was double entered to check the consistency.

### Statistical analyses

The collected data were checked for completeness and entered into Epi-data version 3.1. The data were then exported to IBM SPSS Statistics version 26 for analysis after cleaning. Descriptive statistics like frequency, percentage, mean, and standard deviation (Median and IQR) were used to describe the finding of the study. Bivariate and multivariable logistic regression analyses were used to identify covariates significantly associated with the outcome variables. P-value less than 0.25 and theoretical knowledge were used to include variables in the multivariable logistic regression model. The fitness of the model was checked by Hosmer and Lemeshow’s test. P-value less than 0.05 and adjusted odds ratio with respective 95% confidence interval were used to identify statistically significant covariates.

### Ethics approval and consent to participate

Ethical clearance letter was received from Mettu University College of Health Science’s Ethical Review Committee. Written informed consent was obtained from study participants after explanation of the objective, benefit, and risk of the study. Only those who volunteered were included in the study. Confidentiality was assured by avoiding personal information of the participants and coding questionnaires.

## Results

A total of 402 pregnant women participated in this study giving a response rate of 94.5%.

### Socio-demographic characteristics

Regarding the residence of the respondents, 61.7% were urban and the rest were rural residents. The median age of the respondents was 25 years (IQR = 8). The majority of the respondents (43.5%) were between the ages of 25 and 34 years. More than half of the respondents (52.7%) completed secondary education and above, and 22.6% had no formal education. Majority (81.3%) of them were married and 43.6% of them were housewives. Regarding partner occupation, majority of the women’s partners’ occupation were merchants [Table 2].

**Table 2:** Socio-demographic characteristics of pregnant women in Ilubabor zone, Southwest Ethiopia, February to April 2021 (*n* = 402).

Variables	Category	Frequency	Percent (%)
**Residence**	Rural	154	38.3%
	Urban	248	61.7%
**Age** 25 (IQR = 8)	18–24	155	38.6%
	25–34	175	43.5%
	35 and above	72	17.9%
**Educational status**	No formal education	91	22.6%
	Primary level	99	24.6%
	Secondary level and above	212	52.7%
**Marital status**	Married	327	81.3%
	Separated	32	8.0%
	Divorced	23	5.7%
	Widowed	8	2.0%
	Never married	12	3%
**Occupation**	Housewife	175	43.5%
	Local drink seller	66	16.4%
	Merchant	53	13.2
	Unemployed	12	3.0%
	Others*	96	23.8%
**Partner occupation**	Merchants	134	33.3%
	Farmers	111	27.6%
	Private employee	84	20.9%
	Others*	73	18.2%
**Partner education**	Primary	59	14.9
	Secondary	105	26.5
	College/university	161	40.7
	Read and write	11	2.8
	Illiterate	60	15.2

### The magnitude of delays in maternal health service utilization

A woman stays an average of 1.76 hours (SD = 1.2) to decide to seek health care. The magnitude of the first maternal delay in the study area was 51% (95% CI: 46.0, 56.0). In the study area, the magnitude of the second maternal delay was 48% (95% CI: 43.0, 53.0). A woman travels a median of 1 hour to reach a health facility with IQR = 1.06. A pregnant mother stays 1.13 hours (SD = 0.8) to receive care once she reaches the health facility. The magnitude of the third maternal delay was 33.3% (95% CI: 28.7, 38.2).

### Factors associated with maternal delays

#### Factors associated with delay in deciding to seek care (First maternal delay)

Results from multivariable logistic regression analysis revealed that being unmarried, being unemployed, age, fear of COVID-19, urban residence, and lack of birth preparedness were significantly associated with the first maternal delay. Accordingly, the odds of the first maternal delay was reduced by 85.5% among unmarried pregnant when compared to married pregnant women [AOR (95% CI)], [0.145 (0.046–0.452)], (*p* = .001). Unemployed pregnant women were 4.824 times more likely to encounter the first maternal delay than their employed counterparts [AOR (95% CI)], [4.824 (1.685–13.814)], (*p* = .003). The odds of the first maternal delay was decreased by 77.3% among women above 35 age groups when compared to those who were between 25 and 34 years [AOR (95% CI)], [0.227 (0.089 –.0579)], (*p* = .002). One unit increase in the fear of COVID-19 leads to a 1.112 fold increase in the odds of the first maternal delay [AOR (95% CI)], [1.112 (1.036–1.193)], (*p* = .003). The odds of the first maternal delay was decreased by 48.3% among urban-dwelling mothers than those who were dwelling in rural areas [AOR (95% CI)], [0.517 (0.295–0.909)], (*p* = .022). Pregnant women who did not have birth preparedness were 4.824 times more likely to experience the first maternal delay than those who had birth preparedness [AOR (95% CI)], [6.526 (1.954–21.789)], (*p* = .002) [Table 3].

**Table 3:** Multivariable regression results for factors associated with the first maternal delay among pregnant women in Ilubabor zone, Southwest Ethiopia, February to April 2021 (*n* = 402).

Variables	AOR	95% C.I. for AOR		
		Lower	Upper	p-value
Marital status	0.145	0.046	0.452	0.001
Occupation(employed as ref)	4.824	1.685	13.814	0.003
Educational status	1.080	0.513	2.275	0.839
Age group (15–24)	0.997	0.543	1.832	0.992
Age group(>35)	0.227	.089	.579	0.002
Antenatal care Visit <4	1.467	0.834	2.581	0.183
Fear of COVID-19	1.112	1.036	1.193	0.003
Urban residence	0.517	0.295	0.909	0.022
Know Danger sign	0.691	0.401	1.190	0.183
Not Prepared for birth	6.526	1.954	21.789	0.002

#### Factors associated with delay in reaching health facility (Second maternal delay)

It was found that being unmarried, being unemployed, and age were significantly associated with the second maternal delay. Accordingly, unmarried pregnant women were 5.984 times more likely to encounter the second maternal delay than their married counterparts [AOR (95% CI)], [5.984 (2.930–12.223)], (*p* < .001). Unemployed pregnant women were 26.9 times more likely to experience the second maternal delay than their employed counterparts [AOR (95% CI)], [26.978 (3.477–209.308)], (*p* = .002). The odds of the second maternal delay was decreased by 56.2% among pregnant women who were 35 years and above than those who were between 24 and 35 years [AOR (95% CI)], [0.438 (0.226–0.848)], (*p* = .014) [Table 4].

**Table 4:** Multivariable regression results for factors associated with the second maternal delay among pregnant women in Ilubabor zone, Southwest Ethiopia, February to April 2021 (n = 402).

Variables	AOR	95% C.I. for AOR		p-value
		Lower	Upper	
Marital status	5.984	2.930	12.223	<0.001
Unemployed	26.978	3.477	209.308	0.002
Educational status	1.087	0.586	2.019	0.791
Road to HF present	1.789	0.873	3.668	0.112
Age group (15–24)	1.158	0.698	1.919	0.570
Age group (above 35)	0.438	0.226	0.848	0.014
Means of transport	1.465	0.910	2.361	0.116

#### Factors associated with delay in receiving care (third delay)

Having lengthy admission process and non-spontaneous vaginal delivery were found to be statistically significant determinants for the occurrence of the delay in receiving care in the study area. Accordingly, the odds of third maternal delay was 7.5 times higher among pregnant mothers who had a lengthy admission process than those who had not [AOR (95% CI)], [7.5 (4.053–13.878)], (*p* < .001). Similarly, the odds of the third maternal delay was 1.471 times higher among women delivered with non-spontaneous vaginal delivery than those who delivered with spontaneous vaginal delivery[AOR (95% CI)], [1.471 (1.018–1.999)], (*p* = .012) [Table 5].

**Table 5:** Multivariable regression results for factors associated with the third maternal delay among pregnant women in Ilubabor zone, southwest Ethiopia, February to April 2021(n = 402).

Variables	AOR	95% C.I. for AOR		
		Lower	Upper	p-value
Mode of delivery	1.471	1.018	1.999	0.012
Health provider skilled	0.825	0.400	1.700	0.602
Drugs available at need	0.922	0.257	3.309	0.901
Lengthy admission	7.500	4.053	13.878	<0.001

## Discussion

This study was aimed at examining the magnitude of delays in health service utilization and associated factors among pregnant women in the Ilubabor zone. According to the finding of this study, the magnitude of first maternal delay in the study area is 51% (95% CI: 46.0, 56.0). This finding is similar to the finding of a study conducted in the Gamo zone, Ethiopia, in which the prevalence of first maternal delay was found to be 46.8% [18]. It is, however, higher than the findings from the research conducted in the Hadiya zone and Bahir Dar, Ethiopia, which reported 40.1% and 37.8%, respectively [22, 23]. On the other hand, it is lower than the results of the studies conducted in Bangladesh (69.3%) [24] and Pakistan (71%) [25]. The discrepancy may be attributable to the advancement of maternal health education over time.

The finding of this study also indicates that the magnitude of second maternal delay is 48% (95% CI: 43.0, 53.0). This corresponds to the finding from the study conducted in the Gamo zone, which found 44% [18]. It is, nevertheless, higher than the findings of the studies done in the Hadiya zone and Bahir Dar city, which revealed 29.7% and 31.7%, respectively [22, 23]. The difference may be due to the differences in study time and the global challenging time. On the other hand, it is much lower than the finding of the study conducted in Pakistan (74%) [25]. The difference may be related to improvements in transportation throughout time, as well as attempts being made to reduce maternal mortality.

The study finding also indicates that the magnitude of third maternal delay is 33.3% (95% CI: 28.7, 38.2). This is consistent with the data from studies conducted in the Gamo zone, Hadiya zone, and Bahir Dar, which found the prevalence of third maternal delay to be 31.7%, 32.6%, and 30.7%, respectively [18, 22, 23]. However, it is higher than the prevalence of third maternal delay seen in the research conducted in selected referral hospitals in Ethiopia’s Amhara region and Mozambique, which was 26.9% and 14.2%, respectively [26, 27]. The discrepancy could be explained by differences in study setting and period, as well as the provision of manpower than in the past. In contrast, the magnitude of third maternal delay in this study is substantially lower than data from the research conducted in Yem special woredas, Ethiopia (76.3%) [28], and Pakistan (48%) [25]. This is possibly owed to the difference in sociocultural characteristics of study participants.

This study identified different factors associated with maternal delays. Being unmarried, being unemployed, age, fear of COVID-19, urban residence, and lack of birth preparedness were significantly associated with first maternal delay. According to this study, the odds of the first delay is decreased among unmarried pregnant women when compared to their married counterparts. This could be related to the fact that pregnant women who are not married may decide to seek care on their own, but married pregnant women’s decisions are influenced by their partners. The odds of first maternal delay is higher among unemployed pregnant women compared with those who are employed. This conclusion is supported by the studies conducted in Bahir Dar city and Gamo zone, Ethiopia [18, 23]. Unemployed pregnant women depend on their husband’s income for deciding to seek care, which has an impact on their timely decisions for seeking care. Pregnant women who are 35 years or older are less likely to encounter a first maternal delay than those who are between the ages group of 24 and 35 years. This is corroborated by a Ugandan study which found that younger pregnant women were more likely to experience maternal delay [29]. As the fear of COVID-19 increases by one unit, the odds of experiencing first maternal delay increases by 1.112 fold. The odds of first maternal delay is decreased among urban-dwelling mothers than their rural dwelling counterparts. This is consistent with the research conducted in South Gondar, Ethiopia, and Afghanistan, in which the proportion of delay was higher among rural residents [30, 31]. The odds of first maternal delay is higher among pregnant women who have no birth preparedness than those who are well prepared for institutional delivery. This finding is supported by the study conducted in the Gamo zone, Ethiopia [18].

Similarly, being unmarried, being unemployed, and age were significantly associated with second maternal delay in this study. The odds of second maternal delay is higher among unmarried pregnant women than their married counterparts. The odds of second maternal delay is higher among unemployed pregnant women compared with those employed. It is congruent with research conducted in Bahir Dar in which unemployed mothers had higher odds of maternal delay [23]. Pregnant women who are 35 years or older are less likely to have a second maternal delay than those who are between the ages of 24 and 35 years.

According to the findings of this study, having lengthy admission and non-spontaneous vaginal delivery are significantly associated with the third maternal delay. The odds of the third maternal delay is higher among pregnant mothers who have a lengthy admission process than those who do not have. This finding is supported by the study conducted in the Hadiya zone [22]. The odds of the third maternal delay is higher among women who delivered with non-spontaneous vaginal delivery than those who delivered with spontaneous vaginal delivery. This is supported by the study conducted in Bahir Dar, in which mothers who were not delivered with spontaneous vaginal delivery were about two times more likely to have a third maternal delay than those who delivered in spontaneous vaginal delivery [23].

Although the study assessed access to maternal health services, a very crucial concept for making difference between life and death, during the global challenging time, it is not immune to limitations. *First*, generalizations cannot be made for pregnant women attending delivery at private health facilities. *Second*, the finding may be underestimated because the study did not include women who were severely ill and/or unable to respond and such women may have been greatly impacted by maternal delays. *Third*, the cause-and-effect relationship cannot be established because of the nature of the study.

## Conclusion

This study identified that a significant proportion of mothers experience delays in utilizing maternal health services, although there were no data to suggest exacerbated delays in utilizing maternal health services due to fear of the COVID-19 pandemic. The proportion of maternal delay varies with different factors. Policymakers and the Ministry of Health are urged to make every effort to construct a fully equipped health facility close to mothers’ homes. The need to enhance women’s empowerment to increase their financial autonomy will be beneficial in allowing them to make their own decisions. It is helpful if health facilities increase women’s decision-making capacity and transportation access. Improving admissions processes necessitates immediate intervention. Research that includes private health facilities and/or longitudinal research is encouraged.

## Supporting information

S1 Dataset (SAV)

## Abbreviations

AOR: Adjusted odds ratio
CI: Confidence interval
COVID-19: Coronavirus disease 2019
FCV-19S: Fear of COVID-19 scale
IQR: Interquartile range
JHPIEGO: Johns Hopkins Program for International Education in Gynecology and Obstetrics
SD: Standard deviation
SPSS: Statistical package for social sciences
SSA: Sub-Saharan Africa
